# Association between recent overnight travel and use of long-lasting insecticidal nets in rural Uganda: a prospective cohort study in Tororo

**DOI:** 10.1186/s12936-020-03475-3

**Published:** 2020-11-11

**Authors:** Emmanuel Arinaitwe, Joaniter I. Nankabirwa, Paul Krezanoski, John Rek, Victor Kamya, Adrienne Epstein, Philip J. Rosenthal, Chris Drakeley, Moses R. Kamya, Grant Dorsey, Sarah G. Staedke

**Affiliations:** 1 London School of Hygiene and Tropical Medicine, London, UK; 2 Infectious Diseases Research Collaboration, Kampala, Uganda; 3 Department of Medicine, Makerere University, Kampala, Uganda; 4 Department of Medicine, University of California, San Francisco, CA USA

**Keywords:** Human behaviour, Recent overnight travel, Malaria risk

## Abstract

**Background:**

The burden of malaria in Uganda remains high, but has become increasingly heterogenous following intensified malaria control. Travel within Uganda is recognized as a risk factor for malaria, but behaviours associated with travel are not well-understood. To address this knowledge gap, malaria-relevant behaviours of cohort participants were assessed during travel and at home in Uganda.

**Methods:**

Residents from 80 randomly selected households in Nagongera sub-county, Tororo district were enrolled into a cohort to study malaria in rural Uganda. All participants were given long-lasting insecticidal nets (LLINs) at enrolment and were evaluated every 4 weeks at the study clinic. Participants were asked if they had travelled overnight from their home, and if so, a questionnaire was administered to capture information on travel details and behaviours. Behaviour while travelling was assessed within 4 weeks following travel during the study clinic visit. Behaviour while at home was assessed using a similar questionnaire during two-weekly home visits. Behaviours while travelling *vs* at home were compared using log binomial regression models with generalized estimating equations adjusting for repeated measures in the same individual. Analysis of factors associated with LLIN adherence, such as destination and duration of travel, time to bed during travel, gender and age at time of travel, were assessed using log binomial regression models with generalized estimating equations adjusting for repeated measures in the same individual.

**Results:**

Between October 2017 and October 2019, 527 participants were enrolled and assessed for travel. Of these, 123 (23.2%) reported taking 211 overnight trips; 149 (70.6%) trips were within Tororo. Participants were less likely to use LLINs when travelling than when at home (41.0% vs. 56.2%, relative risk [RR] 0.73, 95% CI 0.60–0.89, p = 0.002); this difference was noted for women (38.8% vs 59.2%, RR 0.66, 95% CI 0.52–0.83, p = 0.001) but not men (48.3% vs 46.6%, RR 0.96, 95% CI 0.67–1.40, p = 0.85). In an adjusted analysis, factors associated with LLIN use when travelling included destination (travelling to districts not receiving indoor residual spraying [IRS] 65.8% vs Tororo district 32.2%, RR 1.80, 95% CI 1.31–2.46, p < 0.001) and duration of travel (> 7 nights 60.3% vs one night 24.4%, RR 1.97, 95% CI 1.07–3.64, p = 0.03).

**Conclusions:**

Travellers, particularly women, were less likely to use LLINs when travelling than when at home. LLIN adherence was higher among those who travelled to non-IRS districts and for more than 1 week, suggesting that perceived malaria risk influences LLIN use. Strategies are needed to raise awareness of the importance of using LLINs while travelling.

## Background

Malaria control interventions have been scaled-up globally, resulting in significant declines in malaria burden [1–3]. Despite these achievements, malaria morbidity remains high worldwide; in 2018, 228 million malaria cases, 93% from Africa, were reported [1]. In Uganda, key malaria control strategies include prompt treatment with artemisinin‐based combination therapy (ACT), universal distribution of long‐lasting insecticidal nets (LLINs), and targeted indoor residual spraying of insecticides (IRS) [2, 4–6]. Although intervention coverage has expanded remarkably in Uganda over the past decade, progress on malaria control has been uneven [7]. According to the 2018–2019 Malaria Indicator Survey, parasite prevalence, measured by microscopy in children under-five, ranged from 0.2% in Kampala to 34.3% in the Karamoja region, and was 3% in Bukedi region, where Tororo district is located and IRS is ongoing [7]. Factors contributing to the heterogeneity of malaria in Uganda include geographical variation in transmission intensity, increasing urbanization, and delivery of IRS to a limited number of districts [8, 9].

Travel is a well-recognized risk factor for malaria [10–18]. Studies from Uganda and elsewhere in Africa have shown that overnight travel is associated with an increased risk of malaria, especially when individuals travel from areas of lower transmission intensity to higher risk areas [19, 20]. A study conducted at three sites of varied malaria transmission in Uganda demonstrated that the incidence of malaria in travellers was over three times higher in the 60 days after overnight travel compared to the 60 days before travelling [19]. Another study in western Uganda found that travelling within the previous 4 weeks from highland areas with low malaria transmission to higher transmission areas was strongly associated with increased malaria risk [20]. Two studies conducted on Bioko Island, Equatorial Guinea, demonstrated that island residents who travelled to the mainland were at increased risk of malaria infection [16, 17]. A survey of Bioko island travellers found that malaria prevalence was significantly higher in passengers returning to the island from the mainland compared to those departing the island [17]. The odds of malaria among Bioko Island residents who travelled was significantly higher than in non-travellers, suggesting that imported malaria cases contributed to the sustained transmission of malaria on the island [16]. Similarly, a study in northern Ethiopia found that travel from high-altitude (low transmission) villages to other areas within the previous month was associated with increased odds of malaria [18]. This evidence suggests that travel within Africa is a risk factor for malaria infection. However, behavioural factors associated with travel that might increase exposure to mosquito vectors, and thus malaria infection, have been less well-explored.

Although travellers may be at increased risk of malaria due to exposure to higher malaria transmission [16, 21], changes in behaviour while away from home may also contribute. Some studies have suggested that individuals who travel within malaria-endemic areas may take part in outdoor activities, go to bed late, and be less likely to use LLINs, all behaviours that increase exposure to mosquitoes and risk of malaria infection [22–25]. To further explore associations between overnight travel and behaviours that might modify the risk of malaria infection, data collected over a two-year period from a cohort of individuals living in Tororo, Uganda, were analysed.

## Methods

### Study site

The study was conducted in Nagongera sub-county in Tororo district, Uganda, a research site for the Program for Resistance, Immunology, Surveillance, and Modelling for malaria in Uganda (PRISM). Details about the site and the two cohort studies led by PRISM (PRISM 1 from 2011 to 2017 and PRISM 2 from 2017 to 2019) have been described elsewhere [26]. Briefly, Nagongera is a rural area with very high malaria transmission, which is now under intensive malaria control. In 2012, the entomological inoculation rate (EIR) in Tororo was 310 infectious bites per person per year [27]. Because of its high malaria burden, Tororo was selected to receive IRS starting in 2015, and to date the district has received seven rounds of IRS (three rounds of the carbamate Bendiocarb, followed by four rounds of the organophosphate pirimiphos-methyl [Actellic]). IRS commenced in December 2014–January 2015 using the carbamate bendiocarb; additional rounds of IRS were delivered in June–July 2015 and November–December 2015. In 2016, the insecticide was changed to the organophosphate pirimiphos-methyl (Actellic) and IRS with Actellic was delivered in June–July 2016, June–July 2017, June–July 2018, and March–April 2019. In addition to IRS, LLINs were distributed to all households in Tororo through national campaigns in 2013 and 2017, in accordance with World Health Organization (WHO) guidelines recommending one LLIN for every two household residents. The interventions have been associated with a drastic reduction in key malaria indicators, including malaria incidence, parasite prevalence and EIR, which was < 1 infectious bite per person per year in 2018 [5, 28, 29].

### Study design and participant enrolment

This study was nested in the PRISM 2 cohort study which has been described in detail elsewhere [26]. Briefly, 6992 households in the study area were enumerated, and out of these, 80 households were randomly selected for cohort participation. Households were included if they met the following selection criteria: (1) at least two household members under 5 years of age, (2) no more than 7 permanent residents, (3) no intention for the household to move from Nagongera sub-county during the study period, and (4) willingness to participate in study follow up activities. These inclusion criteria were designed to ensure that the cohort included a sufficient number of younger children and that the number of household members did not exceed the capacity for participant follow-up.

All members of the enrolled households were screened and enrolled in the cohort study if they met the following selection criteria: (1) full-time resident of the selected household, (2) agreement to come to the study clinic for any illness and scheduled follow up, and (3) provision of written informed consent. Participants were followed up for 2 years, and the cohort was dynamic; any residents that were born into or joined the household were screened for enrolment during the course of the study. Participants were withdrawn from the study if they met the following criteria: (1) permanent movement out of Nagongera sub-county, (2) unable to be located for > 120 days, (3) withdrawal of informed consent, or (4) unable to comply with the study schedule and procedures. All enrolled participants were given LLINs at enrolment and were encouraged to come to the study clinic for all of their medical care.

### Study participant follow up and data collection

Participants were seen at the study clinic monthly for routine follow up. At these visits, participants were asked whether they had travelled overnight since the last visit. A detailed questionnaire was administered to those who travelled to capture data on destination and duration of travel, behavioural factors such as time to bed, and use of LLINs during travel. For malaria prevention, data were collected by asking the following question, “What measures did you take to prevent malaria?”. Responses included; none, slept under LLIN, used mosquito repellents, used mosquito coils, and took anti-malarial. Every 2 weeks, participants were visited at home and the same questionnaire was administered to collect data on behavioural factors while at home.

### Statistical analysis

Data were collected by trained study staff using standardized case record forms and double-entered using Microsoft Access (Microsoft Corporation, Redmond, Washington, USA). All the analyses were performed using Stata, version 14 (Stata Corporation, College Station, Texas, USA). This analysis included data on any overnight travel and behavioural factors collected between October 2017 and October 2019. Overnight travel was defined as travel out of the sub-county of residence and spending at least one night away.

Behavioural factors including adherence to reported LLIN usage the prior night and time to bed were evaluated when study participants were at home and during overnight travel. LLINs use during travel was dichotomized into use most of the time during travel and no use, and time to bed was dichotomized into going to bed before 9 p.m. most of the time and going to bed at 9 p.m. or later mostly. Each overnight trip was paired with the most recent assessment at home as a comparison. For each pair, comparisons between behavioural factors during travel and while at home were made using log binomial regression models with generalized estimating equations adjusting for repeated measures in the same individual, and estimates were reported as relative risks (RR). In addition, an analysis of factors associated with LLIN adherence, such as destination and duration of travel, time to bed during travel, gender and age at time of travel, were also assessed using generalized estimating equations and expressed as relative risks. A *p* value < 0.05 was considered statistically significant.

## Results

### Characteristics of study participants

**Fig. 1:**
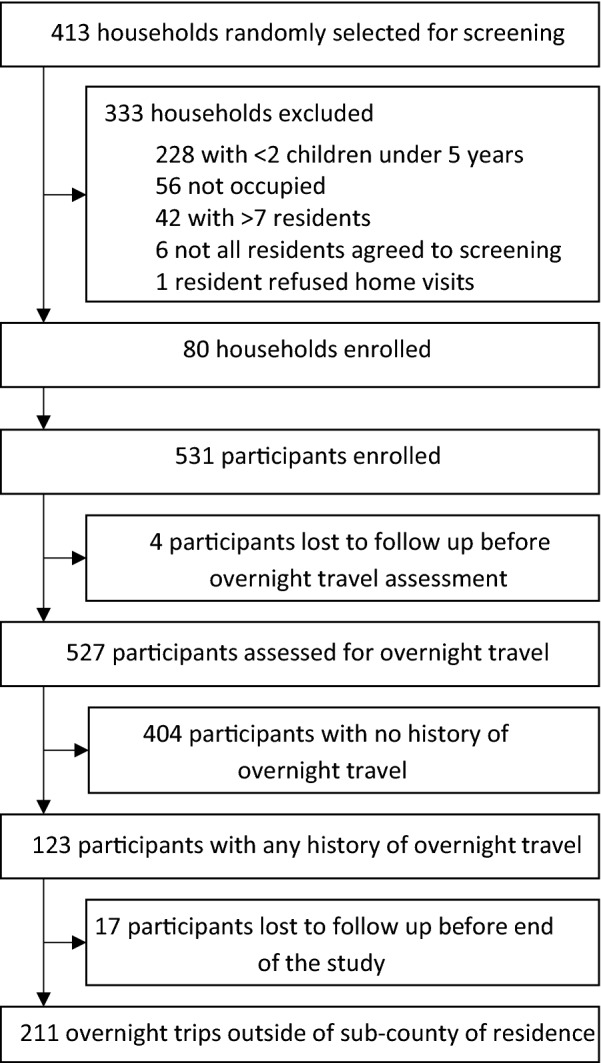
Study profile

**Fig. 2:**
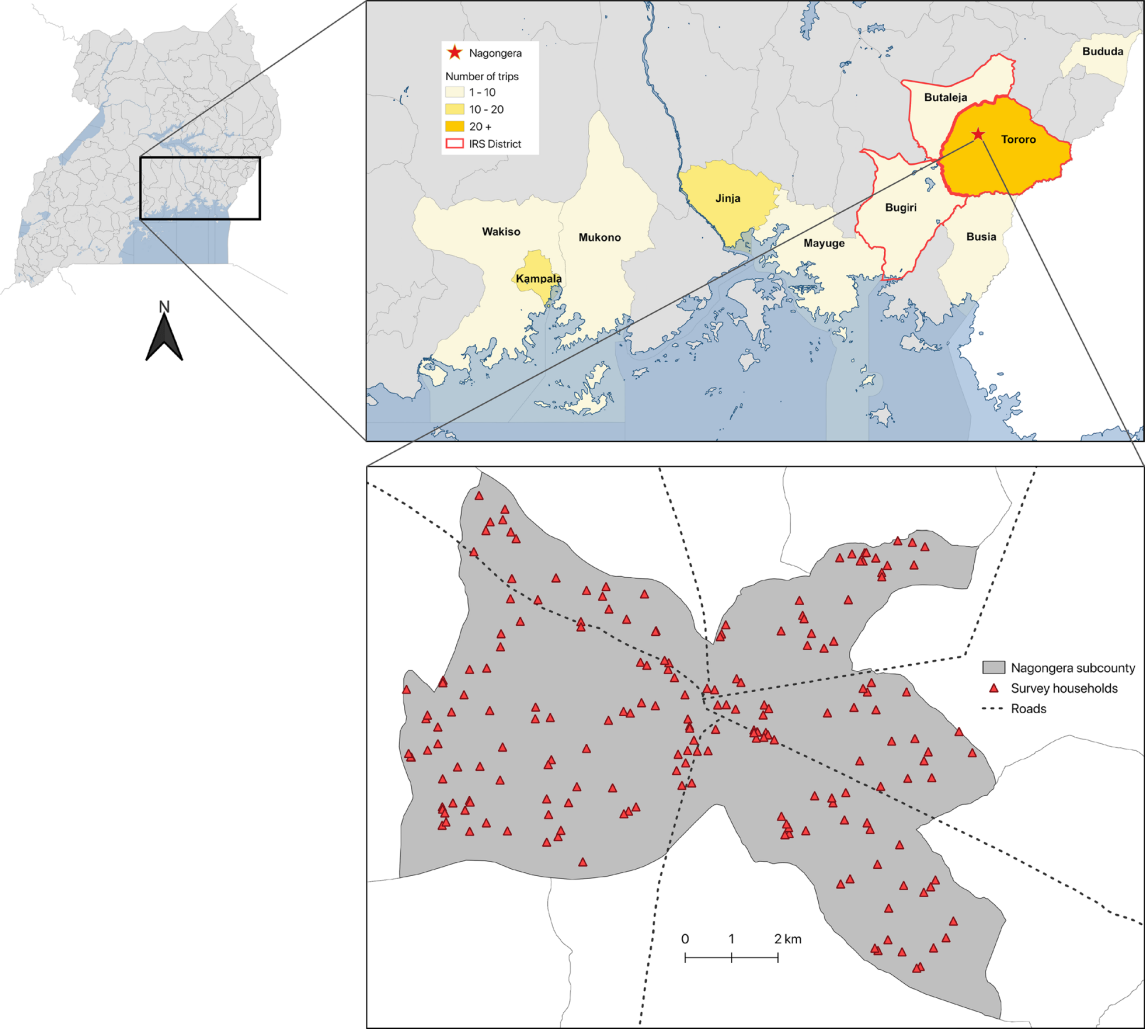
Map of Uganda showing travel destination of study participants to the districts level

From October 2017 through October 2019, all 531 eligible residents from 80 randomly selected households were enrolled; there were no exclusions (Fig. 1). Of these, 527 were assessed for overnight travel. Overall, 123 (23.2%) participants reported at least one overnight trip and were included in the analysis (Table 1). Of these participants who travelled, 65.9% were female. Adults were more likely to travel than children, but school-aged children (5–15 years) were more likely to take longer trips (17 days) than younger children (7 days) or adults (3 days). Most participants travelled short distances (< 30 km) and generally stayed within Tororo district (70.6%). Travel destinations outside Tororo included Bugiri (65 km away) and Butaleja (25 km away), both districts receiving IRS (Fig. 2). Other participants travelled to non-IRS districts (18.0%), and to Kampala (8.1%). The main reason older children and adults travelled was to visit relatives, while children under-five mainly accompanied their parents or guardians. Travellers most commonly stayed with their relatives while away.

**Table 1:** Characteristics of study participants and individual overnight trips

Characteristic	Age categories			
	All ages	< 5 years	5–15 years	> 15 years
Characteristics of study participants^a^				
All participants	531	177	193	161
Female gender, n (% among all participants)	278 (52.4%)	97 (54.8%)	85 (44.0%)	96 (59.6%)
Participants with any overnight trip, n (% total participants)	123 (23.2%)	39 (22.0%)	21 (11.1%)	63 (39.1%)
Female gender, n (% among participants with any travel)	81 (65.9%)	20 (51.3%)	13 (61.9%)	48 (76.2%)
Proportion of travellers from least poor households	43 (35.0%)	10 (25.6%)	7 (33.3%)	26 (41.3%)
Proportion of non-travellers from least poor households	137 (33.6%)	50 (36.2%)	56 (32.6%)	31 (31.6%)
Characteristics of individual overnight trips^b^				
Number of overnight trips	210	53	33	124
Number of trips made by individual participants, n				
1 trip	79	25	17	37
2 trips	18	5	1	12
3 trips	5	2	2	1
4 or more trips	20	3	2	15
Duration of each trip in nights away, median (range)	5 (1-115)	7(1-53)	17 (1-53)	3 (1-115)
Duration of travel categories, n (% total trips)				
1 night	41 (19.5%)	7 (13.2%)	1 (3.0%)	33 (26.6%)
2–3 nights	49 (23.3%)	9 (17.0%)	4 (12.1%)	36 (29.0%)
4–7 nights	43 (20.5%)	11 (20.8%)	6 (18.2%)	26 (21.0%)
More than 7 nights	77 (36.7%)	26 (49.1%)	22 (66.7%)	29 (23.4%)
Destination of travel, n (% total trips)				
Tororo (IRS district)	148 (70.5%)	37 (69.8%)	21 (63.6%)	90 (72.6%)
Other IRS districts	7 (3.3%)	1 (1.9%)	1 (3.0%)	5 (4.0%)
Kampala (no IRS)	17 (8.1%)	5 (9.4%)	7 (21.2%)	5 (4.0%)
Other non-IRS districts	38 (18.1%)	10 (18.9%)	4 (12.1%)	24 (19.4%)
Reason for travel, n (% total trips)				
Visiting relatives	105 (50.0%)	20 (37.7%)	26 (78.8%)	59 (47.6%)
Funeral rite	45 (21.4%)	1 (1.9%)	0	44 (35.5%)
Accompanying parents	39 (18.6%)	32 (60.4%)	7 (21.2%)	0
Caring for the sick	10 (4.8%)	0	0	10 (8.1%)
Business	8 (3.8%)	0	0	8 (6.5%)
Pleasure	2 (1.0%)	0	0	2 (1.6%)
Attending school	1 (0.5%)	0	0	1 (0.8%)
Where participant stayed, n (% total trips)				
Friend/relative’s home	175 (83.3%)	49 (92.5%)	32 (97.0%)	94 (75.8%)
Hospital	13 (6.2%)	2 (3.8%)	1 (3.0%)	10 (8.1%)
Camp or Gardens	22 (10.5%)	2 (3.8%)	0	20 (16.1%)

^a^Based on age at the time of study enrolment

^b^Based on age at the time of travel

### Differences in behaviour during overnight travel versus at home

Overall, LLIN use among cohort participants who travelled was low both at home and while travelling. Participants were significantly less likely to use LLINs when they travelled than when at home (41.0% travel vs. 56.2% home, relative risk (RR) 0.73, 95% CI 0.60–0.89, p = 0.002) (Table 2). However, this difference was modified by gender and age. Trips made by women were less likely to have use of LLINs reported when travelling than when at home (38.8% vs 59.2%; RR 0.66, 95% CI 0.52–0.83, p = 0.001) but no difference in LLIN use was observed for trips made by men (46.6% vs 48.3%; RR 0.96, 95% CI 0.67–1.40, p = 0.85). Stratifying by age, no differences in LLIN use during travel were observed for trips made by younger or school-aged children. However, trips made by older participants (> 15 years) were significantly less likely to have use of LLINs reported when travelling than at home (33.9% vs 61.3%; RR 0.55, 95% CI 0.41–0.74, p < 0.001). Overall, there were no differences in going to bed after 9 pm when travelling versus at home.

**Table 2:** Comparison of behavioural factors at home of residence and during overnight travel

Behavioural factor	Groups	Number of paired observations	Number with a reported behavioural factor (%)		RR (95% CI)	p-value
			At home of residence	During overnight travel		
Sleeping under an LLIN	All	210	118 (56.2%)	86 (41.0%)	0.73 (0.60–0.89)	0.002
	Gender					
	Male	58	28 (48.3%)	27 (46.6%)	0.96 (0.67–1.40)	0.85
	Female	152	90 (59.2%)	59 (38.8%)	0.66 (0.52–0.83)	0.001
	Age					
	< 5 years	53	26 (49.1%)	24 (45.3%)	0.92 (0.62–1.38)	0.70
	5–15 years	33	16 (48.5%)	20 (60.6%)	1.25 (0.86–1.82)	0.25
	> 15 years	124	76 (61.3%)	42 (33.9%)	0.55 (0.41–0.74)	< 0.001
Going to bed after 9 p.m.	All	210	147 (70.0%)	149 (71.0%)	1.01 (0.91–1.13)	0.80
	Gender					
	Male	58	27 (46.6%)	31 (53.5%)	1.15 (0.86–1.53)	0.35
	Female	152	120 (79.0%)	118 (77.6%)	0.98 (0.88–1.10)	0.77
	Age					
	< 5 years	53	15 (28.3%)	20 (37.7%)	1.33 (0.77 2.31)	0.30
	5–15 years	33	23 (69.7%)	19 (57.6%)	0.83 (0.58 1.17)	0.28
	> 15 years	124	109 (87.9%)	110 (88.7%)	1.01 (0.92 1.11)	0.85

### Factors associated with LLIN adherence during overnight travel

Participants who travelled to districts without an IRS program were more likely to sleep under LLINs than those who travelled within Tororo district (65.8% vs 32.2%; RR 1.80, 95% CI 1.31–2.46, p < 0.001) (Table 3). There were no differences in LLIN use when travelling to other destinations (Kampala and other IRS districts). Participants who travelled for more than 7 nights were significantly more likely to use LLINs while travelling than those who travelled for only one night (60.3% vs 24.4%; RR 1.97, 95% CI 1.07–3.64, p = 0.03). Other factors that were not associated with LLIN use during travel included time to bed, gender of the participant, and age at the time of travel.

**Table 3:** Factors associated with LLIN adherence during overnight travel

Factors	Categories	Proportion of trips adherent to LLINs (%)	Univariable analysis		Multivariable analysis	
			RR (95% CI)	p-value	RR (95% CI)	p-value
Destination of travel	Tororo district (IRS)	48/149 (32.2%)	Reference	–	Reference	–
	Other IRS districts	1/7 (14.3%)	0.44 (0.07–2.77)	0.39	0.39 (0.05–2.88)	0.35
	Kampala district	12/17 (70.6%)	2.19 (1.49–3.22)	<0.001	1.49 (0.93–2.40)	0.10
	Other non-IRS districts	25/38 (65.8%)	2.04 (1.47–2.83)	<0.001	1.80 (1.31–2.46)	<0.001
Duration of travel	1 night	10/41 (24.4%)	Reference	–	Reference	–
	2–3 nights	11/49 (22.5%)	0.92 (0.43–1.95)	0.83	0.90 (0.43–1.90)	0.79
	4–7 nights	18/43 (41.9%)	1.72 (0.90–3.27)	0.10	1.44 (0.75–2.80)	0.27
	More than 7 nights	47/78 (60.3%)	2.47 (1.40–4.37)	0.002	1.97 (1.07–3.64)	0.03
Time to bed during travel	Before 9 p.m.	28/62 (45.2%)	Reference	–	Reference	–
	9 p.m. or later	58/149 (38.9%)	0.86 (0.61–1.21)	0.39	1.08 (0.72–1.62)	0.73
Gender	Male	27/58 (46.6%)	Reference	–	Reference	–
	Female	59/153 (38.6%)	0.83 (0.59–1.17)	0.28	1.00 (0.70–1.44)	0.99
Age at time of travel	< 5 years	24/53 (45.3%)	Reference	–	Reference	–
	5–15 years	20/34 (58.8%)	1.30 (0.86–1.96)	0.21	1.16 (0.78–1.73)	0.47
	> 15 years	42/124 (33.9%)	0.75 (0.51–1.10)	0.14	0.87 (0.56–1.37)	0.55

## Discussion

To better understand behavioural factors that might modify the risk of malaria during travel, a cohort of individuals living in Tororo under highly effective malaria control were assessed. Overall, LLIN use in travellers was low, and participants were less likely to use LLINs when they travelled than when at home. However, this finding was true only for women, and adults. Factors associated with higher LLIN use while travelling included travel to non-IRS districts, and travelling for more than 1 week, suggesting that perceived risk of malaria may influence the decision to sleep under an LLIN while away from home.

There are several potential reasons why people may be at increased risk of malaria during travel. In this study, gender differences in LLIN use while travelling were observed. Women were less likely to use LLINs when travelling than at home, but this was not true for men. Interestingly, women reported using LLINs more often than men when at home; however, when travelling, the opposite was true. This suggests that at home, women may be more aware of the importance of sleeping under LLINs to protect against malaria, perhaps reflecting routine distribution of LLINs at antenatal clinics and targeted campaigns to increase LLIN use among pregnant women [30–32]. Some studies carried out in sub-Saharan Africa have evaluated use of LLINs at home and reported increased use among female participants [33–35], but none have assessed gender differences in LLIN adherence during travel. A multi-country analysis of Malaria Indicator Survey and Demographic and Health survey data from 26 countries in Africa, collected between 2011 and 2016, indicated that LLIN use was higher among females aged 15–49 years compared to their male counterparts [34], suggesting that women may be more likely than men to use LLINs when at home. However, when travelling, women may either lack LLINs or the agency to use them, particularly when visiting the home of a friend or relative.

This study found that participants aged 15 years were less likely to use LLINs during travel. Older participants were more likely to travel for funeral rites than younger participants; during such trips, individuals were likely outdoors the entire night. This could partly explain the reduced adherence to LLIN observed in older participants. Findings from a study conducted in four districts in Uganda between March 2012 and January 2013 demonstrated that when individuals travelled for funeral rites and wedding parties, they were less likely to use LLINs [24], suggesting that adults may engage in late night activities that reduce their ability to use LLINs while travelling.

Individuals who travelled to non-IRS districts and those who travelled for more than 7 days were more likely to use LLINs. These findings suggest that the decision to use LLINs may be influenced by destination or duration of travel and the individuals’ perceptions of malaria risk. Indeed, perceptions of malaria risk have been shown to influence the use of LLINs when people travel [22]. In south-eastern Tanzania, in-depth interviews were used to assess perceptions of malaria risk during outdoor and indoor activities. In this study, participants believed that outdoor activities, such as fishing in the river late at night, travelling to farms overnight, and attending parties and funerals held at night, all increased their risk of malaria infection. For situations where use of LLINs was not feasible, participants believed that alternative malaria prevention approaches, including use of mosquito repellents and chemoprophylaxis, were needed.

LLINs are known to reduce malaria morbidity and mortality and are widely used for vector control in Africa [36], but achieving high adherence to LLINs, even at home, is challenging. In this study, just over half of cohort participants who travelled slept under LLINs when at home, despite universal access. Many barriers to LLIN use have been described, including many household members [37, 38], lack of space to hang LLINs [39], lower socioeconomic status, and time since the last LLINs distribution [40]. In this study setting, where malaria transmission dropped substantially, individuals may have felt that it was no longer necessary to use their LLINs [5, 29]. During travel, a possible barrier to LLIN adherence is limited availability of LLINs to use away from home. Mass distribution of LLINs in Uganda follows WHO guidelines, which recommend distributing one LLIN for every two household residents [41]. This may leave no spare LLINs for visitors, or for carrying during travel. In this study, other factors that may have contributed to limited use of LLINs during travel include social barriers, such as attending a funeral or wedding where individuals are expected to stay outdoors all night, or fear of appearing rude or disrespectful during communal gatherings [24]. These factors should be considered when designing strategies to increase LLIN adherence in travellers. In addition, current WHO LLIN distribution recommendations of one LLIN per two household members should be supplemented by encouraging individuals to purchase a spare LLIN for malaria prevention during travel.

A strength of this study is that behaviours at home and during travel within the same individuals were prospectively compared, minimizing the potential for confounding. Similar studies have only assessed malaria-relevant behaviours while travelling, or at home, but not both [25, 42, 43]. A study conducted in south-eastern Tanzania evaluated human behaviour of participants at home [22]. The study found that a high proportion of participants (75%) stayed outdoors in the evenings (between 6 p.m. and 9 p.m.), resulting in exposure to malaria vectors before going to bed. Another study carried out in the Kilombero Valley of Tanzania from November 2015 to March 2016, assessed patterns of behaviour only when travelling, and demonstrated that when individuals travelled for religious, cultural and social gatherings, they stayed outdoors at night till dawn [42]. Previous studies in Uganda that have assessed travel and malaria risk also examined behavioural factors during travel, such as use of LLINs [19, 20]. However, differences in behaviour while travelling versus at home were not explored. The findings from this study suggest that a better understanding of circumstances leading to lower use of LLINs when travelling may be important in guiding malaria prevention measures.

This study had several limitations. First, data on behavioural factors during travel could have been subject to recall bias. However, questionnaires were administered within 4 weeks following travel, and adherence to LLINs at home was assessed every 2 weeks by home visits, to closely evaluate the relationship between behaviours at home and when travelling. Second, the study was conducted in rural Tororo, and few individuals travelled outside of the district. Thus, results may not be generalizable to other settings. Lastly, intensive malaria control with IRS and LLINs resulted in few malaria cases in Tororo. Thus, it was not possible to directly measure the association between behaviours and malaria risk.

## Conclusion

Travel is an important individual risk factor for malaria, and individuals who travel may also threaten malaria control gains, especially in areas on a pathway to elimination. Results from this study suggest that individuals were less likely to use LLINs when travelling. Strategies to increase awareness about the importance of LLIN adherence, particularly in travellers, should be developed and deployed by the National Malaria Control Division of the Ministry of Health, or other stakeholders. Use of LLIN during travel, especially during the holiday season when most people are likely to visit family and friends, should be emphasized. Information on safety of LLINs and appropriate use should be provided over the radio and television, which are common methods of dissemination of information in Uganda. Behavioural Change Communication (BCC) approach should be implemented to help educate people on malaria prevention and proper use of LLINs. Travellers should be encouraged to carry an extra LLIN when travelling, especially when visiting rural areas or those without ongoing IRS. Further research on innovative approaches to prevent malaria in travellers including portable LLINs, effective ways to influence behaviour and increase LLIN use, and acceptability of other malaria prevention measures such as mosquito repellents and chemoprophylaxis, should be encouraged.

## Data Availability

Data and study material are available on request by an e-mail to the corresponding author.
